# Lifespan Volume Trajectories From Non–harmonized T1–Weighted MRI Do Not Differ After Site Correction Based on Traveling Human Phantoms

**DOI:** 10.3389/fneur.2022.826564

**Published:** 2022-05-09

**Authors:** Sarah Treit, Emily Stolz, Julia N. Rickard, Cheryl R. McCreary, Mercedes Bagshawe, Richard Frayne, Catherine Lebel, Derek Emery, Christian Beaulieu

**Affiliations:** ^1^Department of Biomedical Engineering, University of Alberta, Edmonton, AB, Canada; ^2^Departments of Radiology and Clinical Neurosciences, Foothills Medical Centre, Hotchkiss Brain Institute, University of Calgary, Calgary, AB, Canada; ^3^Department of Radiology, Alberta Children's Hospital, University of Calgary, Calgary, AB, Canada

**Keywords:** brain volume, multi–site, MRI, data harmonization, within–subject reliability, reproducibility, healthy lifespan, ComBat

## Abstract

Multi–site imaging consortiums strive to increase participant numbers by pooling data across sites, but scanner related differences can bias results. This study combines data from three research MRI centers, including three different scanner models from two vendors, to examine non–harmonized T1–weighted brain imaging protocols in two cohorts. First, 23 human traveling phantoms were scanned twice each at all three sites (six scans per person; 138 scans total) to quantify within–participant variability of brain volumes (total brain, white matter, gray matter, lateral ventricles, thalamus, caudate, putamen and globus pallidus), and to calculate site–specific correction factors for each structure. Sample size calculations were used to determine the number of traveling phantoms needed to achieve effect sizes for observed differences to help guide future studies. Next, cross–sectional lifespan volume trajectories were examined in 856 healthy participants (5—91 years of age) scanned at these sites. Cross–sectional trajectories of volume versus age for each structure were then compared before and after application of traveling phantom based site–specific correction factors, as well as correction using the open–source method ComBat. Although small systematic differences between sites were observed in the traveling phantom analysis, correction for site using either method had little impact on the lifespan trajectories. Only white matter had small but significant differences in the intercept parameter after ComBat correction (but not traveling phantom based correction), while no other fits differed. This suggests that age–related changes over the lifespan outweigh systematic differences between scanners for volumetric analysis. This work will help guide pooling of multisite datasets as well as meta–analyses of data from non–harmonized protocols.

## Introduction

Multi–site imaging consortiums are becoming an increasingly common strategy to increase power through large participant numbers and to improve generalizability of patient studies. These collaborations have advanced our understanding of numerous disorders, e.g., Alzheimer's disease (ADNI) (1), adolescent mental health (IMAGEN) (2), Autism (ABIDE) (3), and may be particularly valuable when effect sizes are small, as with many neurodevelopmental and psychiatric disorders. However, combining MRI data from multiple sites has the potential to introduce scanner–specific variability, even for relatively robust measures such as brain volume (4–8).

Many studies attempt to prospectively minimize this variability by harmonizing scan protocols across sites (i.e., closely matching acquisition protocols and scanning procedures). This reduces but does not eliminate variability, which inevitably still stems from hardware and software differences (e.g., SNR and image homogeneity from RF coil, calibration of gradient coils for dimensions) (9). Within–participant volume differences from different head coils in the same scanner further emphasizes the impact of hardware (10). Furthermore, prospective harmonization limits a study's ability to use the most optimal protocols (i.e., harmonizing across the lowest capabilities), and is not possible in datasets that are retrospectively combined. Meta–analysis of multiple independent, non–harmonized datasets is of great interest given the ability to achieve participant numbers needed for complex analysis (i.e., correlations with behavior, genetics, etc.). For example, a recent ENIGMA project combined over 80,000 MRI scans from 88 studies to investigate subcortical volume in healthy individuals (11). Going forward, it will remain essential to determine how scanner variability impacts group differences in patient data, changes with age in development or aging studies, and more.

This study examines within–participant reliability of brain volumes across 23 traveling human phantoms, scanned twice at each of three sites (3T Siemens Prisma, 3T GE MR750W and 3T GE MR750), for a total of 138 scans. These data are used to (i) quantify systematic differences in brain volumes between three sites, (ii) determine the number of traveling phantoms needed to detect these site–specific effects; and (iii) determine if correcting for these differences (using two methods) changes the lifespan trajectory of brain volumes assessed in a cross–sectional cohort of 856 healthy individuals (5—91 years of age) scanned across these three sites. Traveling phantom based corrections of lifespan data is compared to correction using the open–source data harmonization method ComBat (12), in order to further determine if traveling phantom sub–studies are useful for multi–site MRI consortiums to pursue.

## Methods

### Participants

This study includes two datasets: (i) a “traveling phantom” cohort and ii) a self–reported healthy lifespan cohort. The traveling phantom dataset consists of 23 healthy adults (29 ± 8 years of age, 20—48 years of age, 10 males) who were scanned twice at each at the University of Alberta (UofA), Alberta Children's Hospital (ACH), and Foothills Medical Center (FMC) for a total of 138 scans (Table 1). The healthy lifespan dataset includes 856 healthy participants (ages 5—91 years, 367 (43%) males) who were scanned once at one of these three sites (Site 1: UofA *n* = 534, Site 2: ACH *n* = 52, Site 3: FMC *n* = 270; Table 1). Healthy development participants were recruited separately at each site as part of independent studies of typical brain development and/or aging (13, 14), through advertising and word of mouth, and were screened for psychiatric, neurological and developmental disorders as well as contraindications to MRI. Traveling phantom participants were recruited locally through word of mouth and were all familiar with undergoing MRI scans to ensure compliance (i.e., mostly graduate students from Edmonton and Calgary). All participants (or their guardian) provided written informed consent prior to participating in this study.

**Table 1:** Participant demographics.

		**Total**	**Site 1 – University of Alberta (UofA)**	**Site 2 – Alberta Children's Hospital (ACH)**	**Site 3 – Foothills Medical Center (FMC)**
Traveling phantom dataset					
	Scans	138	46	46	46
	Scans per person	6	2	2	2
	*N*	23^a^			
	Age (years)	29 ± 8^a^			
	Sex (M/F)	10/13^a^			
Healthy development dataset					
	*N* ^b^	856	534	52	270
	Age (years)	39 ± 22 (5—91)	35 ± 22 (5—90)	11 ± 3 (6—17)	51 ± 17 (18—91)
	Sex (M/F)	367/489	231/303	27/25	109/181

^a^*The same 23 participants were scanned at each site*.

^b^*One scan per participant, separate participants at each site*.

### Image Acquisition

**Figure 1:**
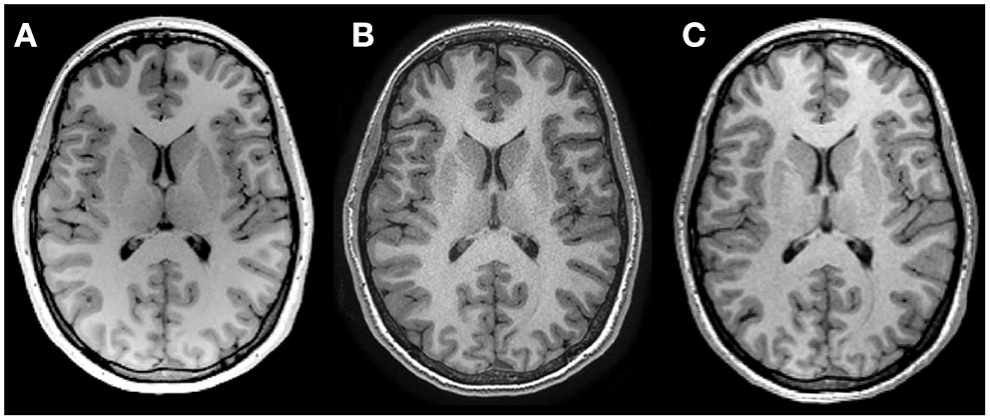
T1 weighted images of the same 27–year–old female scanned at **(A)** Site 1 University of Alberta, **(B)** Site 2 Alberta Children's Hospital and **(C)** Site 3 Foothills Medical Center, using non–harmonized protocols. See Table 2 for acquisition protocols.

MRI protocols were not harmonized across sites but were instead consistent with existing protocols for the system located at each center being used in concurrent but independent ongoing development/aging studies. Traveling phantoms were scanned with the same protocol as the healthy lifespan cohort at each site to systematically evaluate within and between site differences in brain volumes. Scans were collected on (i) 3T Siemens Prisma with 64 channel coil, sagittal MPRAGE, 0.87 × 0.87 × 0.85 mm^3^, TE/TR/TI = 2.4/1800/900 ms, 3:39 min at Site 1 – UofA; (ii) 3T GE MR750W with 32 channel coil, axial IR–SPGR, 0.8 mm isotropic, TE/TR/TI = 3.2/8.2/600 ms, 5:38 min at Site 2 – ACH; and (iii) 3T GE MR750 with a 12–channel coil, sagittal IR–SPGR, 1 mm isotropic, TE/TR/TI = 2.6/6.3/650 ms, 5:44 min at Site 3— FMC (14). Table 2 summarizes the protocols and Figure 1 presents a representative set of T1–weighted images of a single axial slice from a traveling participant at all three sites.

**Table 2:** Scan protocols (optimized independently at each site).

	**Site 1 – University of Alberta (UofA)**	**Site 2 – Alberta Children's Hospital (ACH)**	**Site 3 – Foothills Medical Center (FMC)**
Model	3T Siemens Prisma	3T GE MR750W	3T GE MR750
Head RF coil	64–channel	32–channel	12–channel
Sequence	MPRAGE	IR–SPGR	IR–SPGR
Resolution (mm^3^)	0.87 × 0.87 × 0.85	0.8 × 0.8 × 0.8	1.0 × 1.0 × 1.0
Slices	208	226	166
TE/TR/TI (ms)	2.4/1800/900	3.2/8.2/600	2.6/6.3/650
Acquisition plane	Sagittal	Axial	Sagittal
Acquisition time (min: sec)	3:39	5:38	5:44

### Image Analysis and Statistics

All images (for both traveling phantom and healthy development scans from all sites) were processed in volBrain (15) to yield total brain, white matter, total gray matter (cortical and deep gray matter), lateral ventricle, thalamus, caudate, putamen, and globus pallidus volumes (left and right combined).

The percent difference between consecutive within–participant scans at each site was calculated as a measure of the test-retest reliability of each scanner. Reliability of volume measurements across sites was assessed with intraclass correlation coefficients (ICC) and Repeated Measures ANOVA (with pairwise comparisons between sites), after averaging each participants' consecutive scans within a site. Coefficient of variation (CV) within participants across all three sites, and between participants at each site were also calculated. Given that the number of traveling human phantoms included in this study (*n* = 23) was chosen to well exceed the typical number used in past literature, e.g., *n* = 2—10 (6, 16, 17), one aim here was to determine the number required to detect site related differences in future studies. To this end, sample size calculations were performed in G^*^Power (version 3.1, IDRE Stats) using partial eta squared values from RM–ANOVA to determine the number of traveling human phantoms needed to detect observed effects of site on volume measurements for each structure.

Site–specific traveling phantom–based correction factors (TP–correction) were calculated by using the participant mean volume for each site divided by the grand mean for that participant (average volume across all six scans). This percent difference was then averaged across all 23 participants per structure for each site and used to correct the absolute volumes of the healthy development cohort for systematic site difference using a ratio multiplier. For example, if one site was found to have an average of 2% higher volumes for a given structure compared to the grand mean, then the individual volumes for that structure for all scans from that site were multiplied by 1/1.02. As an alternative to traveling–phantom based correction, raw lifespan data was also corrected using ComBat (12) which employs an empirical Bayes method for correction of site effects (18). This analysis was run in Python and included sex and age as covariates (https://github.com/Jfortin1/ComBatHarmonization).

Lifespan volume trajectories in the main dataset were estimated with linear, quadratic, cubic and exponential curves; significant curves with the lowest AIC values were considered the best fit (Supplementary Table 1), calculated with the “statsmodels” module in Python. Raw data was then corrected for site (as described above), and the previously chosen best fit curves were fit to TP–corrected and ComBat–corrected data. Differences in the age and intercept parameters between fits in TP–corrected and ComBat corrected data (relative to raw data) were tested with *Z* statistics. In addition, correlations between volumes given by TP–correction vs. ComBat were tested with Pearson's correlations. For the purposes of this manuscript, volumes of males and females were combined into a single fit vs. age.

Given that 62% of the lifespan sample was scanned at UofA (thus dominating the fits), a post–hoc analysis was performed to repeat curve fitting in a subset of participants that included only half of the sample from Site 1 UofA (*n* = 267, age 5—90 with the same age distribution as the total sample) in addition to all of the scans from Site 2 ACH (*n* = 52) and Site 3 FMC (*n* = 270) for a total of 589 participants. *Z*–tests were used to test for differences in fit parameters between raw data, TP–corrected and ComBat corrected data in this subset. Note that ComBat correction was run separately in this cohort given that ComBat cannot be run ad–hoc on single subjects.

## Results

### Traveling Phantoms

**Figure 2:**
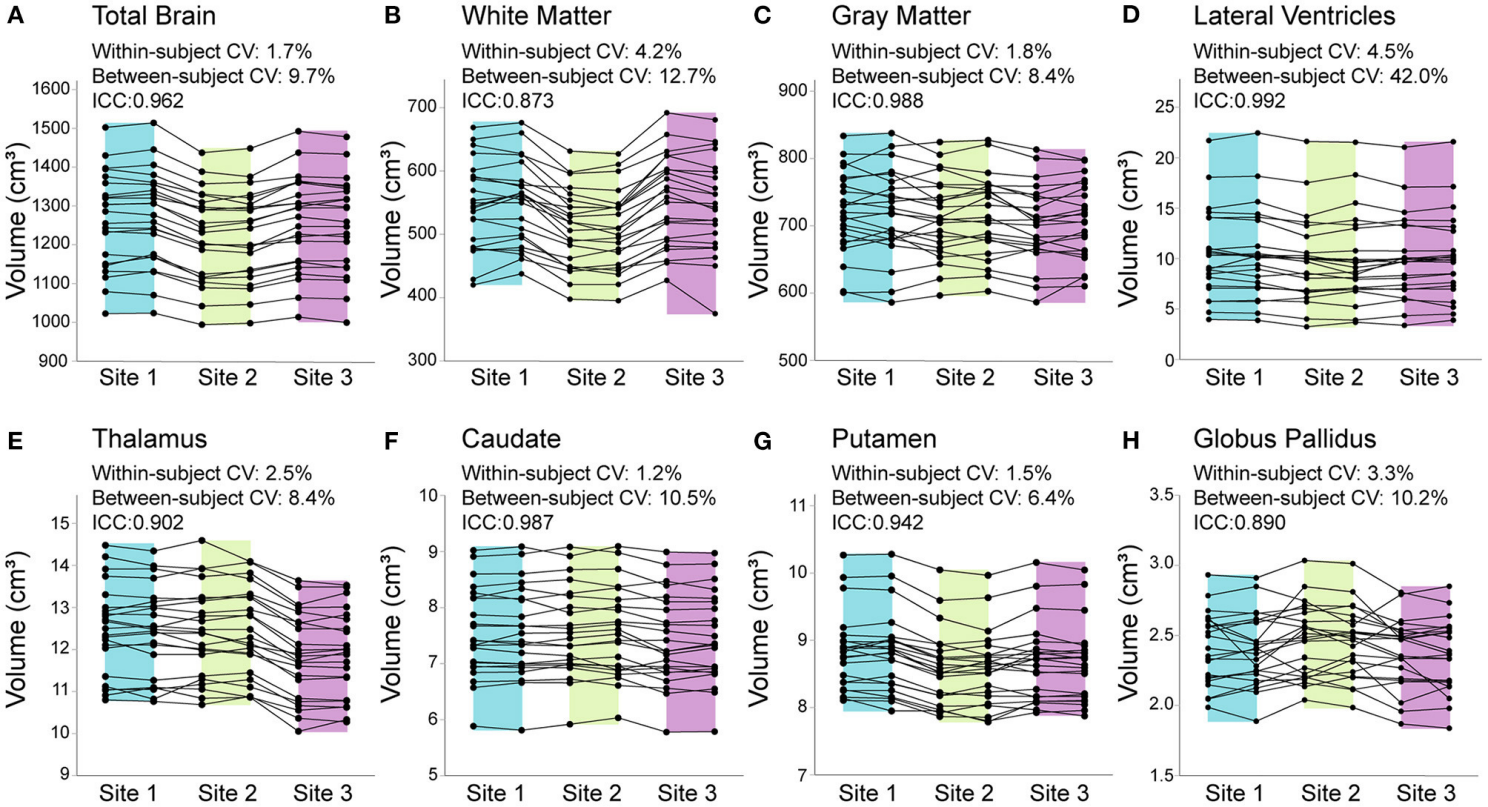
Total brain **(A)**, white matter **(B)**, gray matter **(C)**, lateral ventricle **(D)**, thalamus **(E)**, caudate **(F)**, putamen **(G)**, and globus pallidus **(H)** volumes from all 23 traveling phantom participants scanned twice each at each of the 3 sites (138 datapoints per structure total). Percent change in consecutive scans within subject at each site was <1% for all structures (at all sites), except lateral ventricle and white matter volume at Site 3 (FMC), which had 1% and 1.6% difference (respectively) between test–retest scans. Coefficient of variation (CV) within–participants across the 3 sites ranged from 1.2% [**(F)** caudate] to 4.5% [**(D)** lateral ventricles], while between–participant CV was much larger for all structures, ranging from 6.4% [**(G)** putamen] to 42% [**(D)** lateral ventricles]. Intraclass correlation coefficients (ICCs) suggested excellent agreement between scanners, ranging from 0.873 [**(B)** white matter] to 0.992 [**(D)** lateral ventricles]. Shading indicates the range for each site (Site 1/UofA – blue; Site 2/ACH – green; Site 3/FMC – purple).

**Figure 3:**
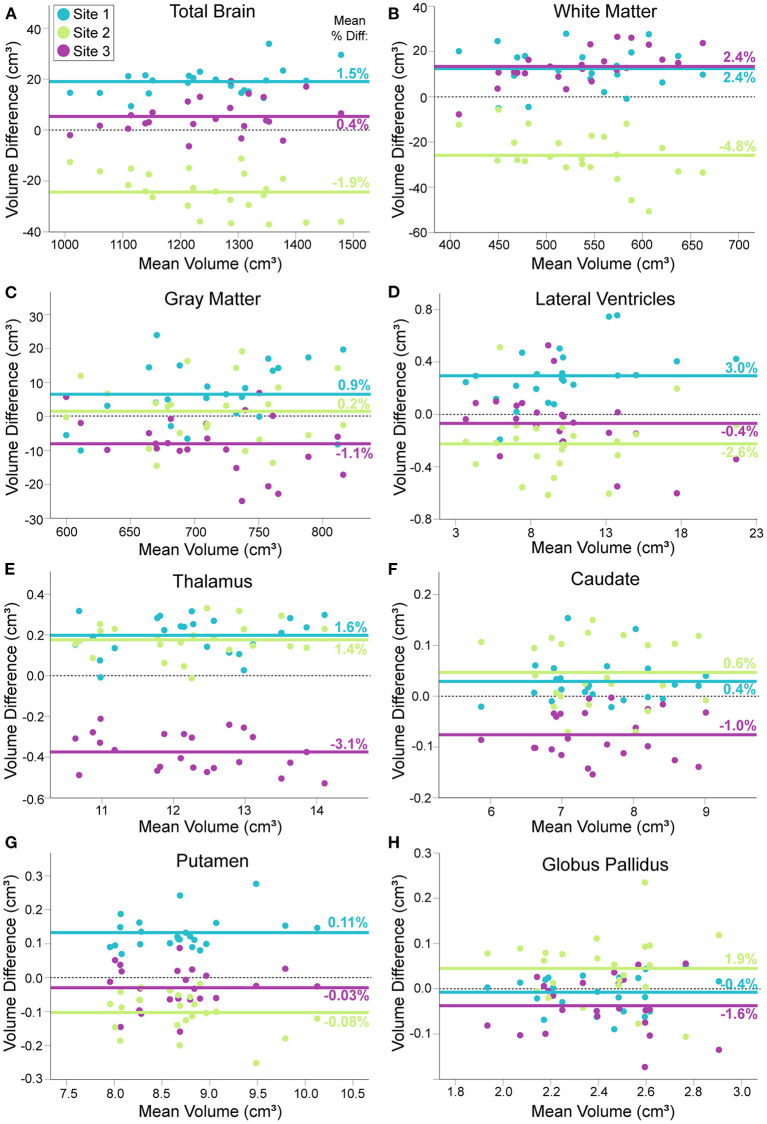
Modified Bland–Altman plots, showing the mean difference between each traveling phantom's average volume at a given site (two scans) and their individual average across all three sites (Y axis), plotted against their mean volume over all three sites (X axis). For total brain **(A)**, white matter **(B)**, gray matter **(C)**, lateral ventricle **(D)**, thalamus **(E)**, caudate **(F)**, putamen **(G)** and globus pallidus volumes **(H)**. Each group of three points per volume (aligned vertically) represents one of the 23 traveling human phantoms. Solid lines indicate the mean volume difference across participants for each site (mean percent difference indicated numerically on each line); dotted black line indicates a mean difference of zero for reference. Some structures show close alignment between two sites [e.g., white matter **(B)**, thalamus **(G)**] while others suggest that one site is close to the grand mean while the other two sites differ [e.g. total brain volume **(A)**]. Plots indicate volume difference between sites does not depend on mean volume, with the exception of total brain and white matter volume at Site 2 (ACH), which becomes slightly more different from the total mean at larger volumes.

Percent differences in consecutive scans within site (i.e., test–retest for each scanner) was <1% for all structures at all sites, except lateral ventricle volume (1%) and white matter volume (1.6%) at Site 3. Within–participant CVs across all three sites ranged from 1.2—4.5% across structures (Figure 2). In all structures, between participant variability in the 23 young adults was two to nine times larger than within participant variability across all three scanners. ICC values ranged from 0.873 (white matter) to 0.992 (lateral ventricles), suggesting excellent overall consistency across participants between scanners. Nonetheless, RM–ANOVA was significant for all structures, suggesting small systematic differences between sites (Table 3; Figures 2, 3). For example, thalamus volume was ~3% lower at Site 3 (FMC) compared to the other two sites, while total brain volume and white matter volume were ~2% and ~5% lower, respectively, at Site 2 (ACH) relative to the other two sites. Sample size calculations yielded a range of 3 to 10 traveling phantoms needed to detect these differences (Table 3). Figure 3 further demonstrates that site effects appear independent of structure volume, with the exception of white matter volume at Site 2 (ACH) which appears to deviate further from the mean at larger volumes.

**Table 3:** Repeated measures ANOVA effects of site on traveling phantom volumes.

	**Omnibus Effect of Site**			**Sample required to detect site effect**	**Pairwise comparisons^a^**		
	**(RM–ANOVA)**						
	***F***	***p*–value**	**Partial η^2^**		**UofA vs. ACH**	**UofA vs. FMC**	**ACH vs. FMC**
Total brain	159.3	<0.001	0.879	3	<0.001	<0.001	<0.001
White matter	89.0	<0.001	0.802	3	<0.001	NS	<0.001
Gray matter	10.0	<0.001	0.313	8	NS	<0.001	0.021
Lateral ventricles	19.2	<0.001	0.466	5	<0.001	<0.001	NS
Thalamus	196.9	<0.001	0.900	3	NS	<0.001	<0.001
Caudate	23.7	<0.001	0.519	5	NS	<0.001	<0.001
Putamen	67.8	<0.001	0.755	3	<0.001	<0.001	0.013
Globus pallidus	7.53	0.002	0.256	10	NS	NS	0.015

^a^*Bonferroni corrected for multiple comparisons. NS, not significant*.

### Lifespan Trajectories Before and After Site Correction

**Figure 4:**
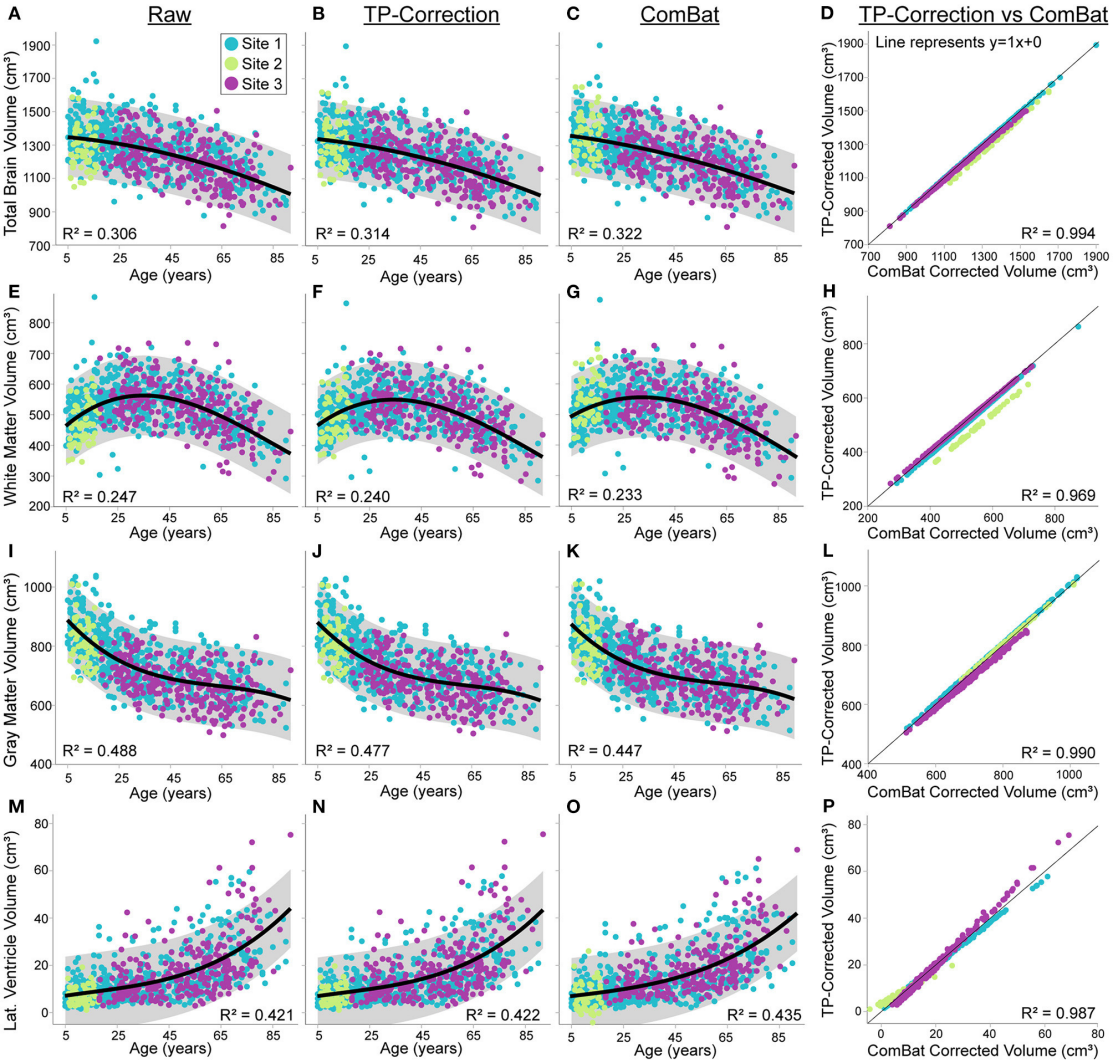
Total brain, white matter, gray matter and lateral ventricle volume across the lifespan in 856 healthy participants (5—91 years of age) scanned at the three sites [Site 1 (UofA) – blue, Site 2 (ACH) – light green, Site 3 (FMC) – purple] before (column 1) and after traveling phantom based correction (column 2) or ComBat correction (column 3). Gray shaded regions indicate the 95% confidence interval for each fit. Column 4 indicates that volumes after traveling phantom (TP) based correction and ComBat based correction are highly correlated, but site–specific differences are observable particularly for white matter and lateral ventricle volumes. Specifically, the correlation between traveling phantom corrected and ComBat corrected volumes at Site 2 (ACH) is right shifted for white matter, and has a shallower slope for lateral ventricles (suggesting an interaction with age). Trajectories and correlations are shown for total brain **(A–D)**, white matter **(E–H)**, gray matter **(I–L)** and lateral ventricle volumes **(M–P)**.

**Figure 5:**
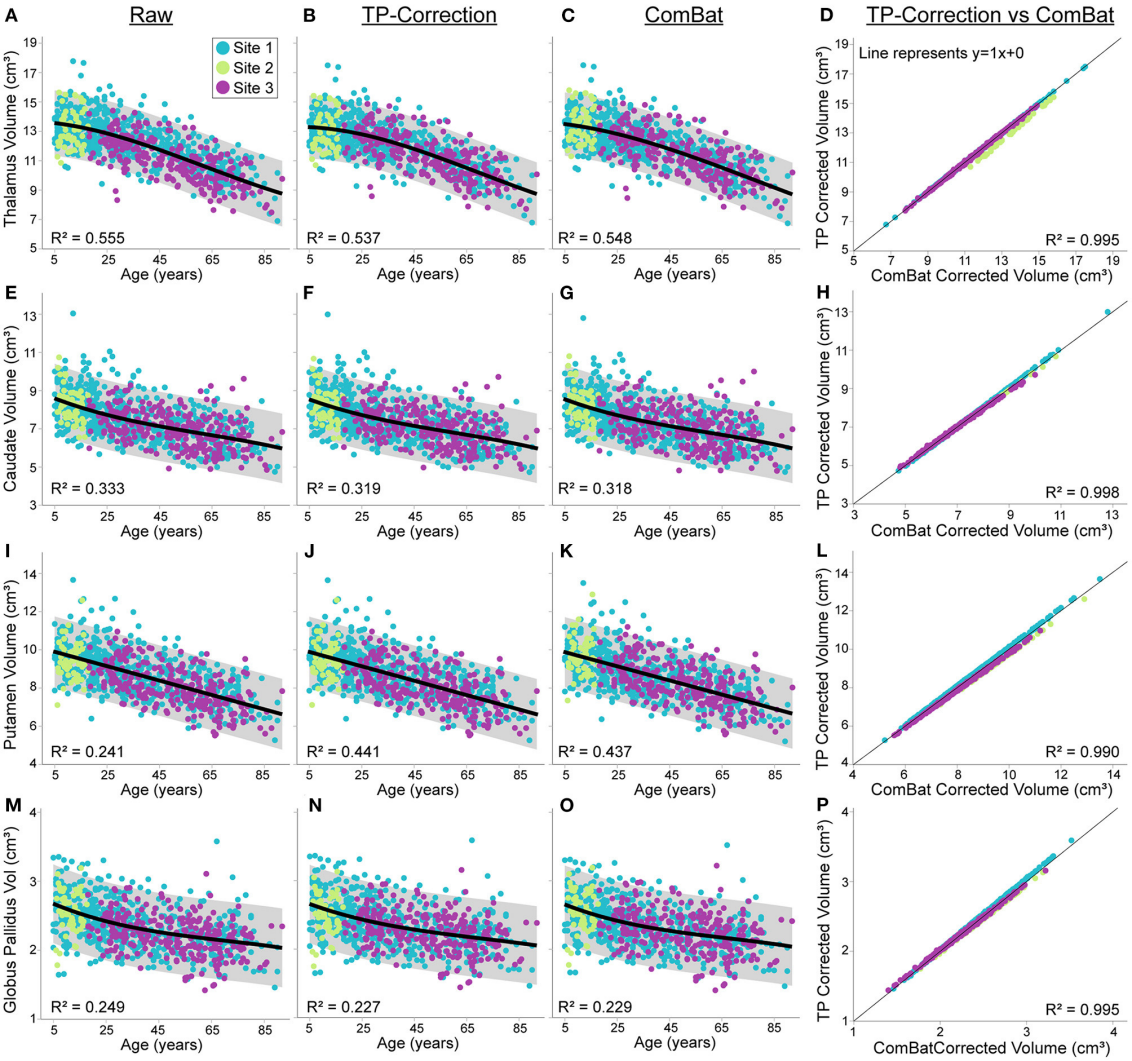
Thalamus, caudate, putamen and globus pallidus volume trajectories in 856 healthy participants (5—91 years of age) scanned at the three sites [Site 1 (UofA) – blue, Site 2 – (ACH) – light green, Site 3 (FMC) – purple] before (column 1) and after traveling phantom based (column 2) or ComBat site correction (column 3). All volumes were lower at older ages, following either linear (putamen) or cubic trajectories (caudate, thalamus and globus pallidus). Gray shaded areas represent the 95% confidence interval of each fit. Traveling phantom corrected volumes and ComBat corrected volumes were highly correlated in all structure, following a similar pattern at all 3 sites (column 4). Trajectories and correlations are shown for thalamus **(A–D)**, caudate **(E–H)**, putamen **(I–L)**, and globus pallidus volumes **(M–P)**.

The first analyses here are on the uncorrected raw volumes vs. age (column 1 of Figures 4, 5). Total brain volume was best fit to a quadratic trajectory, with older ages associated with lower volumes (Figure 4A). White matter volume best fit a cubic trajectory that peaked around 30 years of age (after which older ages were associated with smaller white matter volumes) (Figure 4E). Both total gray matter and lateral ventricle volume fit cubic trajectories, demonstrating that older ages are associated with smaller total gray matter volumes (steeper drop from 5—25 years) and larger lateral ventricle volumes (steeper rise after ~ 60 years) (Figures 4I,M). Thalamus, caudate, putamen and globus pallidus volumes all demonstrated a negative relationship with age following either a linear (putamen) or cubic trajectory (thalamus, caudate, globus pallidus – Figures 5A,E,I,M; Table 4). The fit for thalamus volume trajectory is relatively flat at younger ages, and then decreases steeply with increasing age, which may reflect an early upswing in volume in very early childhood not captured here. In contrast, caudate and globus pallidus volume fits decrease more steeply at younger ages, continuing more gradually at older ages.

**Table 4:** Volume versus age fit parameters in uncorrected (raw) and corrected (TP correction, ComBat) data.

		**Age**	**Age^**2**^**	**Age^**3**^**	**Constant**
		**β (SE)**	**β (SE)**	**β (SE)**	**β (SE)**
Total brain	Raw	−1.15 (0.80)	−0.03 (9.24E−3)		1354 (14)
	TP Correction	−1.46 (0.78)	−0.02 (9.05E−3)		1346 (14)
	ComBat	−1.91 (0.79)	−0.02 (9.13E−3)		1368 (14)
White matter	Raw	8.64 (1.15)	−0.16 (2.97E−2)	5.97E−4 (2.25E−4)	425 (12)
	TP Correction	7.45 (1.12)	−0.13 (2.89E−2)	4.82E−4 (2.19E−4)	432 (12)
	ComBat	5.84 (1.16)	−0.12 (2.99E−2)	3.33E−4 (2.26E−4)	**468 (12)**
Gray matter	Raw	−10.77 (1.21)	0.15 (3.13E−2)	−8.07E−4 (2.38E−4)	938 (13)
	TP Correction	−10.49 (1.19)	0.15 (3.08E−2)	−8.13E−4 (2.34E−4)	929 (13)
	ComBat	−10.05 (1.19)	0.15 (3.08E−2)	−8.11E−4 (2.34E−4)	921 (13)
Lateral ventricles	Raw	0.19 (0.15)	−2.53E−3 (3.76E−3)	5.4E−5 (2.90E−5)	6.24 (1.56)
	TP Correction	0.18 (0.14)	−2.38E−3 (3.72E−3)	5.3E−5 (2.80E−5)	6.16 (1.54)
	ComBat	0.14 (0.14)	−5.48E−4 (3.65E−3)	3.6E−5 (2.80E−5)	6.34 (1.52)
Thalamus	Raw	−1.26E−2 (1.96E−2)	−8.73E−4 (5.07E−4)	5.00E−6 (4.00E−6)	13.64 (0.21)
	TP Correction	1.70E−3 (1.89E−2)	−9.93E−4 (4.89E−4)	5.00E−6 (4.00E−6)	13.30 (0.20)
	ComBat	−1.60E−2 (1.90E−2)	−6.59E−4 (4.91E−4)	3.00E−6 (4.00E−6)	13.59 (0.20)
Caudate	Raw	−5.78E−2 (1.60E−2)	5.66E−4 (4.13E−4)	−3.00E−6 (3.00E−6)	8.87 (0.17)
	TP Correction	−5.43E−2 (1.60E−2)	5.26E−4 (4.13E−4)	−3.00E−6 (3.00E−6)	8.80 (0.17)
	ComBat	−5.61E−2 (1.60E−2)	5.66E−4 (4.14E−4)	−3.00E−6 (3.00E−6)	8.81 (0.17)
Putamen	Raw	−3.77E−2 (1.45E−3)			10.09 (0.06)
	TP Correction	−3.76E−2 (1.45E−3)			10.08 (0.06)
	ComBat	−3.71E−2 (1.44E−3)			10.07 (0.06)
Globus pallidus	Raw	−1.76E−2 (5.02E−3)	1.87E−4 (1.30E−4)	−8.85E−7 (9.84E−7)	2.76 (0.05)
	TP Correction	−1.53E−2 (5.05E−3)	1.50E−4 (1.30E−4)	−7.05E−7 (9.89E−7)	2.73 (0.05)
	ComBat	−1.66E−2 (5.02E−3)	1.81E−4 (1.30E−4)	−9.0E−7 (9.84E−7)	2.73 (0.05)

*Significant differences in Z tests between TP -corrected or ComBat corrected parameters (vs. uncorrected) are shown in bold (p < 0.05). TP, Traveling Phantom*.

**Figure 6:**
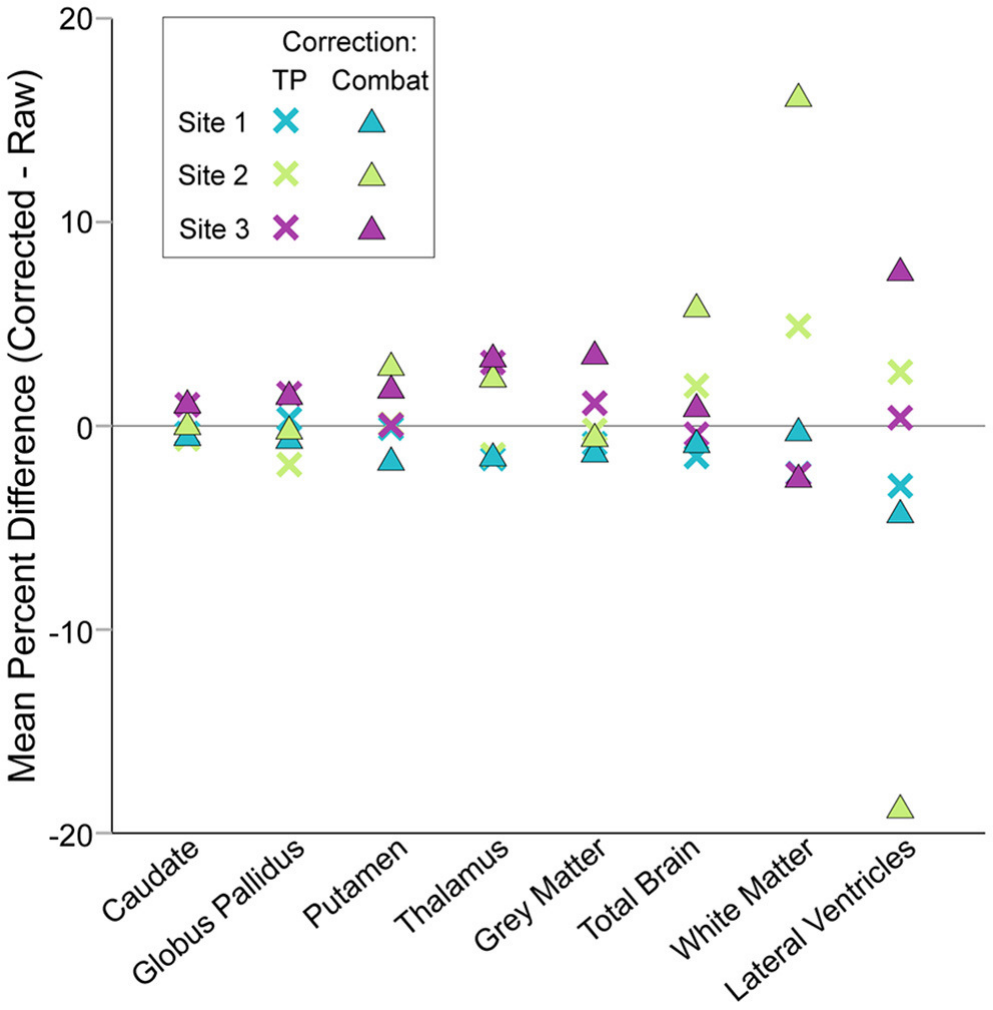
Mean percent changes between corrected and raw volumes by site, structure and correction method. Traveling phantom and ComBat correction methods yielded similar results for deep gray matter structures, resulting in small magnitude changes in volumes. Conversely, changes in lateral ventricle, white matter and total brain volumes after ComBat correction were larger than after traveling phantom based correction. Note that negative values for lateral ventricle volume produced by ComBat correction were excluded prior to calculation of mean percent difference here.

Application of traveling phantom based correction factors did not change the parameters of any fits, and had little impact on *R*^2^ values (Table 4; Figures 4, 5, column 2). ComBat correction yielded similar volume–age trajectories for all structures (Figures 4, 5, column 3), but did result in small but significant change in the intercept parameter for white matter (Table 4). There were no significant differences in fit parameters for any other structure. As expected, TP–corrected and ComBat corrected volumes were highly correlated for all structures *(R*^2^ = 0.969 – 0.998) (Figures 4, 5, column 4). However, slight deviations can be seen between sites, e.g., white matter values are right–shifted to greater volumes for ComBat correction relative to TP–correction at Site 2 (ACH) (Figure 4H). Differences in the magnitude of correction between these two methods is also appreciable when examining mean percent change for each structure (Figure 6) which likewise indicates larger magnitude corrections from ComBat for white matter and lateral ventricle volume, particularly for Site 2 (ACH). A *post–hoc* analysis including only half of the sample from Site 1 UofA (n = 267, age 5—90 years) along with the full sample from the other two sites revealed that with the exception of the caudate and putamen, all structures fit the same trajectories in the subsample (*n* = 589) as in the full sample (*n* = 856) (Supplementary Table 1). The caudate was best fit to a cubic trajectory in the full sample and a quadratic trajectory in the subsample, while the putamen was best fit to a linear trajectory in the full sample and a cubic in the subsample, albeit both with very similar AIC values between models (Supplementary Table 1). In this smaller subsample, the only fit parameters with significant differences before and after correction were age and intercept parameters for white matter, which were significantly different between the raw and ComBat corrected data (Supplementary Table 2).

## Discussion

This study demonstrated small systematic variations of brain volume (within–participant differences of 1—5%) across three 3T MRI scanners (two vendors – Siemens and GE with two different models), measured in 23 healthy adult traveling phantoms. ICC values ranged from 0.873 (white matter) to 0.992 (lateral ventricles), suggesting excellent overall consistency between scanners. These values are on par or higher than ICC values reported in past volumetric traveling phantom studies (4, 5, 19). Both here and in Pfefferbaum et al. (19), the lateral ventricles had the highest ICC value. Although this stems in part from stable within–participant measurements, it also likely reflects the high between participant variability observed for this structure, even within traveling phantoms of a similar age. Despite identifying some systematic multi–site differences, traveling phantom based correction did not significantly change volume vs. age fit parameters for any structure in a sample of 856 healthy volunteers, ages 5—91 years scanned at these three sites. ComBat correction changed the raw data slightly more than traveling phantom based correction for some structures (and specifically for one site) but only yielded significant albeit small changes in lifespan fit parameters for white matter, with no changes in lifespan trajectories for any other structure. This finding suggests that changes in volume with age outweigh smaller systematic scanner acquisition differences. This holds promise for meta–analysis development/aging studies that combine data across scanners and may not have access to traveling phantom data for site correction.

Volume measurement variability between scanners can stem from numerous factors, including differences in hardware (vendor, field strength, gradient coils, RF coil, etc.), pulse sequence software and image contrast, and analysis pipelines (e.g., *via* automated detection of structural boundaries). Image quality differences may also vary by site based on scanning procedures, an observation that may be particularly true for younger and older participants where motion may be more common (20, 21). Effect of scanner can also differ by structure, which may be attributable to structure size (22), or differences in scanner–dependent image contrast that may influence segmentation of some structures more than others. Here we observed small systematic reductions of white matter volume at one site (Site 2 – ACH) in our traveling human phantoms. Evaluation of segmentations in the traveling phantom data suggests that this stems from differences in white matter labeling within the deep gray matter regions that likely results from differences in T1–weighted image contrast. Proximity to the lateral ventricles (e.g., caudate) may confer stability relative to structures bordering white matter (e.g., lateral border of the thalamus) that rely more heavily on scanner–dependent gray–white matter contrast. Lower thalamus volumes at Site 3 (FMC) may result from the larger voxel size and reduced contrast, particularly along the lateral border (Figure 1).

Some studies attempt to prospectively mitigate this variability by harmonizing acquisition protocols across sites. Although this approach has many advantages, it does not completely eliminate site effects, and frequently results in sub–optimal image acquisition when sites are matched to the lowest performance of the scanner cohorts. Several past studies have used traveling human phantoms to then quantify site–related bias in volumetric measures in multi–site datasets; however, this approach is limited by the cost and effort associated with sending control participants to each site. As a result, most of these studies have included limited traveling participants [e.g., one—-to three participants; (6, 17, 23)], while some have included up to 6—10 participants [e.g., (4, 5, 16)]. One previous study of voxel–based morphometry included 32 participants each scanned twice on two scanners (24), though the scans were collected one year apart on two scanners in the same center (i.e., the study did not require travel). Given the range of participants used in the literature and the common goal of reducing costs associated with reproducibility tests for future studies, sample size calculations were used here to determine the number of traveling phantoms required to detect between site differences for various brain volumes, which suggested a need for between 3 and 10 participants (varying by structure, Table 3). This is in line with a previous study that estimated a minimum of 6 traveling phantoms needed to perform traveling phantom based correction of brain volumes (25). Modalities with more inherent sources of variability (e.g., diffusion imaging, quantitative susceptibility mapping) or that combine data collected across scanners of varying field strengths are likely to require a greater number of traveling phantoms (26–29). However, for the latter, previous volumetric studies have concluded that the effect of age far outweighs the effect of vendor or field strength (30, 31).

Given the increasing interest in retrospective data pooling across sites, as undertaken by large meta–analysis studies such as ENIGMA (32) or LIFESPAN (33), many studies attempt to reduce inter–site variability by applying specific post–processing methods [e.g., ComBat – used here; (25, 34)] or by including site as a statistical covariate [e.g., (35)], while others do not account for site at all [e.g., (36)]. There is currently no consensus on the best approach, which likely depends on the sample size of the data, the expected effect size of the results, and other study specific factors. Here we find minimal effects of ComBat correction on the lifespan trajectories of brain volumes; however, the raw data from each site was already highly overlapped despite significant site effects demonstrated in our traveling phantom study. Previous studies demonstrating that ComBat correction increases statistical significance of group effects (34) or strengthens associations with age (12) may have had larger site effects in their raw data, given these studies combined data over 33 and 11 scanners, respectively. A smaller study examining test–retest reliability of volume measurements in 20 traveling phantom subjects scanned at two sites found that ComBat reduced measurement bias less than traveling phantom based corrections (25). Indeed, the degree to which ComBat decreases inter–subject variability and increases statistical power likely depends on the magnitude of site effects in the underlying raw data.

Non–linear fitting of lifespan data can be influenced by many factors and must be interpreted with caution (37, 38). General additive models or local polynomial regression may provide more accurate estimates for data over this age span, but the advantage of using more discrete fits is the ability to compare parameters, here used for the purpose of quantifying change after traveling phantom and ComBat correction. The overall pattern of relationships with age here on 856 self–reported healthy participants 5—91 years was similar to previous work, despite differences in age spans, fitting models and subject numbers across the literature in this area. Trajectories of white matter volume increased with age, plateaued and decreased thereafter, lateral ventricle volume increased with age (though most notably starting in the 50s and 60s), while all other structure volumes decreased at varying rates. Discrepancies in volume trajectories during childhood are commonly found across studies, particularly for structures that have been modeled to have early life increases of volume. For example, thalamus volume has been shown to have a positive association with age during childhood in some cross–sectional work [e.g. (39, 40)] while longitudinal work finds mixed results in samples starting at 5 years of age (40–42). Here we do not model an increase but rather a plateau at the earliest ages followed by steeper decreases at older ages, which may suggest an earlier increase that is already leveling off by age 5. A recent cross–sectional study of over 80,000 volunteers from 3—90 years models an upswing of volume in the thalamus that peaks around 10 years of age (11), highlighting the need for lifespan samples with volunteers younger than 5, and suggesting that increases may not be well captured in samples starting closer to this peak.

Several limitations of this study should be acknowledged. Intraclass correlation coefficient and RM–ANOVA may be overly simplistic relative to more complex statistical methods like hierarchical clustering methods [e.g., (43)]; however, they nonetheless provide intuitive and quantitative measures to facilitate comparison. Only one segmentation protocol was used here (volBrain) despite known differences between various brain structure segmentation algorithms (44, 45). However, volBrain has been shown to yield superior dice coefficients to Freesurfer and FIRST when segmentations are compared to gold–standard manual segmentation (15), and the volBrain pipeline includes advanced denoising and tissue–based intensity normalization, which the authors suggest improves the consistency of signal to noise and contrast thus reducing the impact of site effects in combined datasets (15, 37). Volume–age trajectories were not fit separately in males and females to reduce the complexity of analysis and the number of comparisons here, at the cost of increased inter–subject variability given known differences in volumes between males and females.

Site–specific corrections were calculated based on within participant means across the three sites, without knowledge of the ground truth (i.e., which scanner yields the most accurate measure of true volume). Absolute volumes can be assessed with calibrated resolution phantoms such as used in ADNI (46), however this was not available for our study. Scaling corrections were used rather than absolute volume corrections (i.e., shifting) given the wide age span (and therefore volume span) of this lifespan dataset; however, this assumes that scanner related bias scales with size. ComBat has a distinct advantage of being widely available and free (relative to time consuming and costly collection of traveling phantom data), but is limited by being dataset–specific (i.e., cannot be applied to a single scan) and can also yield implausible results (e.g., producing negative values for lateral ventricle volume for a handful of subjects here). Regardless, this study suggests that correction with either method yields similar results, neither of which substantially influence lifespan trajectories. Thus, intrasubject variability and change with age outweigh both scanner related differences and changes produced by either harmonization method.

These results hold promise for the feasibility of multi–site meta–analysis studies of volume based on automated segmentation of 3D T1–weighted images at 3T to advance our understanding of brain development, aging and disease through pooling of very large participant numbers.

## Data Availability Statement

The raw data supporting the conclusions of this article will be made available by the authors, without undue reservation.

## Ethics Statement

The studies involving human participants were reviewed and approved by Health Research Ethics Boards of the University of Alberta and University of Calgary. Written informed consent to participate in this study was provided by the participant, or their parent/legal guardian for participants under the age of 18.

## Author Contributions

ST contributed to study design, data collection, statistical analysis, created figures, and wrote the manuscript. ES, JR, CM, and MB carried out data collection and processing. CM, CL, RF, DE, and CB designed the study, oversaw all aspects of data collection and analysis, and edited the manuscript. All authors contributed to the article and approved the submitted version.

## Funding

Operating funds for the University of Alberta cohort were provided by the University of Alberta Hospital Foundation, Women's and Children's Health Research Institute, and Canadian Institutes for Health Research. Data collection at the Alberta Children's Hospital was funded by the Natural Sciences and Engineering Research Council. Data collection at the Foothills Medical Center was supported by Canadian Institutes of Health Research and the Seaman Family MR Research Center. Salary support was provided by the Canada Research Chairs Program (CB and CL) and the Hopewell Professorship (RF).

## Conflict of Interest

The authors declare that the research was conducted in the absence of any commercial or financial relationships that could be construed as a potential conflict of interest.

## Publisher's Note

All claims expressed in this article are solely those of the authors and do not necessarily represent those of their affiliated organizations, or those of the publisher, the editors and the reviewers. Any product that may be evaluated in this article, or claim that may be made by its manufacturer, is not guaranteed or endorsed by the publisher.

## References

[1] Mueller SG Weiner MW Thal LJ Petersen RC Jack CR Jagust W. Ways toward an early diagnosis in Alzheimer's disease: the Alzheimer's Disease Neuroimaging Initiative (ADNI). Alzheimers Dement. (2005) 1:55–66. 10.1016/j.jalz.2005.06.003 17476317 PMC1864941

[2] Schumann G Loth E Banaschewski T Barbot A Barker G Büchel C. The IMAGEN study: reinforcement–related behaviour in normal brain function and psychopathology. Mol Psychiatry. (2010) 15:1128–39. 10.1038/mp.2010.4 21102431

[3] Di Martino A Yan CG Li Q Denio E Castellanos FX Alaerts K. The autism brain imaging data exchange: towards a large–scale evaluation of the intrinsic brain architecture in autism. Mol Psychiatry. (2014) 19:659–67. 10.1038/mp.2013.78 23774715 PMC4162310

[4] Cannon TD Sun F McEwen SJ Papademetris X He G van Erp TGM. Reliability of neuroanatomical measurements in a multisite longitudinal study of youth at risk for psychosis. Hum Brain Mapp. (2014) 35:2424–34. 10.1002/hbm.22338 23982962 PMC3843968

[5] Huang L Wang X Baliki MN Wang L Apkarian AV Parrish TB. reproducibility of structural, resting–state BOLD and DTI Data between identical scanners. Plos ONE. (2012) 7. 10.1371/journal.pone.0047684 PMC3485040 23133518

[6] Gouttard S Styner M Prastawa M Piven J Gerig G. Assessment of reliability of multi–site neuroimaging via traveling phantom study. Med Image Comput Comput Assist Interv. (2008). 11(Pt 2): 263–70. 18982614 10.1007/978-3-540-85990-1_32 PMC2758043

[7] Jovicich J Marizzoni M Sala–Llonch R Bosch B Bartres–Faz D Arnold J. Brain morphometry reproducibility in multi–center 3 T MRI studies: a comparison of cross–sectional and longitudinal segmentations. Neuroimage. (2013) 83:472–84. 10.1016/j.neuroimage.2013.05.007 23668971

[8] Cercignani M Bammer R Sormani MP Fazekas F Filippi M. Inter–sequence and inter–imaging unit variability of diffusion tensor MR imaging histogram–derived metrics of the brain in healthy volunteers. ANJR Am J Neuroradiol. (2003) 24:638–43. 12695195 PMC8148683

[9] Voelker MN Kraff O Brenner D Wollrab A Weinberger O Berger MC. The traveling heads: multicenter brain imaging at 7 Tesla. Magma. (2016) 29:399–415. 10.1007/s10334-016-0541-8 27097904

[10] Panman JL To YY van der Ende EL Poos JM Jiskoot LC Meeter LHH. Bias Introduced by Multiple Head Coils in MRI Research: an 8 Channel and 32 Channel Coil Comparison. Front Neurosci. (2019) 13:729. 10.3389/fnins.2019.00729 31379483 PMC6648353

[11] Dima D Modabbernia A Papachristou E Doucet GE Agartz I Aghajani M. Subcortical volumes across the lifespan: Data from 18,605 healthy individuals aged 3–90 years. Hum Brain Mapp. (2021) 43:452–69 10.1002/hbm.25320 33570244 PMC8675429

[12] Fortin JP Cullen N Sheline YI Taylor WD Aselcioglu I Cook PA. Harmonization of cortical thickness measurements across scanners and sites. Neuroimage. (2018) 167:104–20. 10.1016/j.neuroimage.2017.11.024 29155184 PMC5845848

[13] Treit S Rickard JN Stolz E Solar K Seres P Emery D. A normative brain MRI database of neurotypical participants from 5 to 90 years of age. Can J Neurol Sci. (2022). 10.1017/cjn.2021.513. [Epub ahead of print]. 34974849

[14] McCreary CR Salluzzi M Andersen LB Gobbi D Lauzon L Saad F. Calgary normative study: design of a prospective longitudinal study to characterise potential quantitative MR biomarkers of neurodegeneration over the adult lifespan. BMJ Open. (2020) 10:e038120. 10.1136/bmjopen-2020-038120 32792445 PMC7430487

[15] Manjón JV Coupé P. volBrain: An online MRI brain volumetry system. Front Neuroinform. (2016) 10:30. 10.3389/fninf.2016.00030 27512372 PMC4961698

[16] West J Blystad I Engström M Warntjes JB Lundberg P. Application of quantitative MRI for brain tissue segmentation at 1. 5 T and 30 T field strengths. PLoS ONE. (2013) 8:e74795. 10.1371/journal.pone.0074795 24066153 PMC3774721

[17] Chalavi S Simmons A Dijkstra H Barker GJ Reinders AATS. Quantitative and qualitative assessment of structural magnetic resonance imaging data in a two–center study. BMC Med Imaging. (2012) 12:27 10.1186/1471-2342-12-27 22867031 PMC3447701

[18] Johnson WE Li C Rabinovic A. Adjusting batch effects in microarray expression data using empirical Bayes methods. Biostatistics. (2007) 8:118–27. 10.1093/biostatistics/kxj037 16632515

[19] Pfefferbaum A Rohlfing T Rosenbloom MJ Sullivan EV. Combining atlas–based parcellation of regional brain data acquired across scanners at 1. 5 T and 30 T field strengths. Neuroimage. (2012) 60:940–51. 10.1016/j.neuroimage.2012.01.092 22297204 PMC3303927

[20] Savalia NK Agres PF Chan MY Feczko EJ Kennedy KM Wig GS. Motion–related artifacts in structural brain images revealed with independent estimates of in–scanner head motion. Hum Brain Mapp. (2017) 38:472–92. 10.1002/hbm.23397 27634551 PMC5217095

[21] Roalf DR Quarmley M Elliott MA Satterthwaite TD Vandekar SN Ruparel K. The impact of quality assurance assessment on diffusion tensor imaging outcomes in a large–scale population–based cohort. Neuroimage. (2016) 125:903–19. 10.1016/j.neuroimage.2015.10.068 26520775 PMC4753778

[22] Jovicich J Czanner S Han X Salat D van der Kouwe A Quinn B. MRI–derived measurements of human subcortical, ventricular and intracranial brain volumes: Reliability effects of scan sessions, acquisition sequences, data analyses, scanner upgrade, scanner vendors and field strengths. Neuroimage. (2009) 46:177–92. 10.1016/j.neuroimage.2009.02.010 19233293 PMC2866077

[23] Lebel C Mattson SN Riley EP Jones KL Adnams CM May PA. A longitudinal study of the long–term consequences of drinking during pregnancy: heavy in utero alcohol exposure disrupts the normal processes of brain development. J Neurosci. (2012) 32:15243–51. 10.1523/JNEUROSCI.1161-12.2012 23115162 PMC3515671

[24] Takao H Hayashi N Ohtomo K. Effects of study design in multi–scanner voxel–based morphometry studies. Neuroimage. (2014) 84:133–40. 10.1016/j.neuroimage.2013.08.046 23994315

[25] Maikusa N Zhu Y Uematsu A Yamashita A Saotome K Okada N. Comparison of traveling–subject and ComBat harmonization methods for assessing structural brain characteristics. Hum Brain Mapp. (2021) 42:5278–87. 10.1002/hbm.25615 34402132 PMC8519865

[26] Pinto MS Paolella R Billiet T Van Dyck P Guns PJ Jeurissen B. harmonization of brain diffusion MRI: concepts and methods. Front Neurosci. (2020) 14:396. 10.3389/fnins.2020.00396 32435181 PMC7218137

[27] Fox RJ Sakaie K Lee JC Debbins JP Liu Y Arnold DL. a validation study of multicenter diffusion tensor imaging: reliability of fractional anisotropy and diffusivity values. Am J Neuroradiol. (2012) 33:695–700. 10.3174/ajnr.A2844 22173748 PMC8050446

[28] Magnotta VA Matsui JT Liu D Johnson HJ Long JD Bolster BD. Jr.. Multicenter reliability of diffusion tensor imaging. Brain connect. (2012) 2:345–55. 10.1089/brain.2012.0112 23075313 PMC3623569

[29] Takao H Hayashi N Kabasawa H Ohtomo K. Effect of scanner in longitudinal diffusion tensor imaging studies. Hum Brain Mapp. (2012) 33:466–77. 10.1002/hbm.21225 21391276 PMC6869949

[30] Potvin O Mouiha A Dieumegarde L Duchesne S. Normative data for subcortical regional volumes over the lifetime of the adult human brain. Neuroimage. (2016) 137:9–20. 10.1016/j.neuroimage.2016.05.016 27165761

[31] Potvin O Dieumegarde L Duchesne S. Normative morphometric data for cerebral cortical areas over the lifetime of the adult human brain. Neuroimage. (2017) 156:315–39. 10.1016/j.neuroimage.2017.05.019 28512057

[32] Thompson PM Stein JL Medland SE Hibar DP Vasquez AA Renteria ME. The ENIGMA Consortium: large–scale collaborative analyses of neuroimaging and genetic data. Brain Imaging Behav. (2014) 8:153–82. 10.1007/s11682-013-9269-5 24399358 PMC4008818

[33] Pomponio R Erus G Habes M Doshi J Srinivasan D Mamourian E. Harmonization of large MRI datasets for the analysis of brain imaging patterns throughout the lifespan. Neuroimage. (2020) 208:116450. 10.1016/j.neuroimage.2019.116450 31821869 PMC6980790

[34] Radua J Vieta E Shinohara R Kochunov P Quidé Y Green MJ. Increased power by harmonizing structural MRI site differences with the ComBat batch adjustment method in ENIGMA. Neuroimage. (2020) 218:116956. 10.1016/j.neuroimage.2020.116956 32470572 PMC7524039

[35] Fennema–Notestine C Gamst AC Quinn BT Pacheco J Jernigan TL Thal L. Feasibility of multi–site clinical structural neuroimaging studies of aging using legacy data. Neuroinformatics. (2007) 5:235–45. 10.1007/s12021-007-9003-9 17999200

[36] Wang Y Xu Q Luo J Hu M Zuo C. Effects of age and sex on subcortical volumes. Front Aging Neurosci. (2019) 11:259. 10.3389/fnagi.2019.00259 31616285 PMC6775221

[37] Coupé P Catheline G Lanuza E Manjón JV. Towards a unified analysis of brain maturation and aging across the entire lifespan: a MRI analysis. Hum Brain Mapp. (2017) 38:5501–18. 10.1002/hbm.23743 28737295 PMC6866824

[38] Fjell AM Walhovd KB Westlye LT Ostby Y Tamnes CK Jernigan TL. When does brain aging accelerate? Dangers of quadratic fits in cross–sectional studies. Neuroimage. (2010) 50:1376–83. 10.1016/j.neuroimage.2010.01.061 20109562

[39] Brain Development Cooperative Group. Total and Regional brain volumes in a population–based normative sample from 4 to 18 years: the nih mri study of normal brain development. Cereb Cortex. (2011) 22:1–12. 10.1093/cercor/bhr018 21613470 PMC3236790

[40] Narvacan K Treit S Camicioli R Martin W Beaulieu C. Evolution of deep gray matter volume across the human lifespan. Hum Brain Mapp. (2017) 38:3771–90. 10.1002/hbm.23604 28548250 PMC6867004

[41] Tamnes CK Walhovd KB Dale AM Ostby Y Grydeland H Richardson G. Brain development and aging: Overlapping and unique patterns of change. Neuroimage. (2013) 68:63–74. 10.1016/j.neuroimage.2012.11.039 23246860 PMC5378867

[42] Raznahan A Shaw PW Lerch JP Clasen LS Greenstein D Berman R. Longitudinal four–dimensional mapping of subcortical anatomy in human development. Proc Natl Acad Sci U S A. (2014) 111:1592–7. 10.1073/pnas.1316911111 24474784 PMC3910572

[43] Hawco C Viviano JD Chavez S Dickie EW Calarco N Kochunov P. A longitudinal human phantom reliability study of multi–center T1–weighted, DTI, and resting state fMRI data. Psychiatry Res Neuroimaging. (2018) 282:134–42. 10.1016/j.pscychresns.2018.06.004 29945740 PMC6482446

[44] Dadar M Duchesne S. Reliability assessment of tissue classification algorithms for multi–center and multi–scanner data. Neuroimage. (2020) 217:116928. 10.1016/j.neuroimage.2020.116928 32413463

[45] Liu S Hou B Zhang Y Lin T Fan X You H. Inter–scanner reproducibility of brain volumetry: influence of automated brain segmentation software. BMC Neurosci. (2020) 21:35. 10.1186/s12868-020-00585-1 32887546 PMC7472704

[46] Gunter JL Bernstein MA Borowski BJ Ward CP Britson PJ Felmlee JP. Measurement of MRI scanner performance with the ADNI phantom. Med Phys. (2009) 36:2193–205. 10.1118/1.3116776 19610308 PMC2754942

