# Supplementary material for: Lifespan Volume Trajectories From Non–harmonized T1–Weighted MRI Do Not Differ After Site Correction Based on Traveling Human Phantoms
Source: Front Neurol. 2022 May 9;13:826564. doi: 10.3389/fneur.2022.826564 (PMC9124864; doi:10.3389/fneur.2022.826564)
Supplement: Supplementary file 1 [file Data_Sheet_1.docx]

**Supplementary Materials Table 1:** AIC values from curve-fitting of uncorrected data.

|  | **Linear** | **Quadratic** | **Cubic** | **Exponential** |
| --- | --- | --- | --- | --- |
| Full dataset (n=856) | | | | |
| Total Brain | 10626 | 10618 | 10620 | 10630 |
| White Matter | 9809 | 9605 | 9598 | 9811 |
| Grey Matter | 9777 | 9700 | 9689 | 9761 |
| Lateral Ventricles | 6115 | 6066 | 6063 | 6068 |
| Caudate | 2294 | 2288 | 2287 | 2290 |
| Putamen | 2319 | 2321 | 2323 | 2322 |
| Thalamus | 2647 | 2638 | 2636 | 2659 |
| Globus Pallidus | 317.3 | 306.8 | 305.9 | 314.1 |
| Subset (n=589) | | | | |
| Total Brain | 7321 | 7313 | 7313 | 7324 |
| White Matter | 6777 | 6629 | 6625 | 6779 |
| Grey Matter | 6730 | 6675 | 6672 | 6719 |
| Lateral Ventricles | 4225 | 4180 | 4171 | 4181 |
| Caudate | 1572 | 1570 | 1570 | 1571 |
| Putamen | 1610 | 1609 | 1608 | 1613 |
| Thalamus | 1827 | 1823 | 1822 | 1833 |
| Globus Pallidus | 215.9 | 213.6 | 213.1 | 214.8 |

**Supplementary Materials Table 2:** Fitting parameters in uncorrected (raw) and corrected (TP corrected, ComBat corrected) data. Significant differences in fitting parameters denoted in bold.

|  |  | **Age** | **Age^2^** | **Age^3^** | **Constant** |
| --- | --- | --- | --- | --- | --- |
|  |  | **β (SE)** | **Β (SE)** | **β (SE)** | **β (SE)** |
| Total Brain | Raw | -0.76 (0.96) | -3.15E-2 (1.11E-2) |  | 1336 (18) |
|  | TP Correction | -1.30 (0.95) | -2.58E-2 (1.09E-2) |  | 1337 (17) |
|  | ComBat | -1.82 (0.95) | -2.13E-2 (1.10E-2) |  | 1357 (17) |
| White Matter | Raw | 8.82 (1.42) | -0.15 (3.64E-2) | 5.50E-4 (2.73E-4) | 416 (15) |
|  | TP Correction | 7.07 (1.38) | -0.12 (3.54E-2) | 3.79E-4 (2.66E-4) | 433 (15) |
|  | ComBat | **4.21 (1.43)** | -7.18E-2 (3.67E-2) | 1.04E-4 (2.76E-4) | **487 (16)** |
| Grey Matter | Raw | -9.34 (1.48) | 0.11 (3.78E-2) | -5.00E-4 (2.85E-4) | 918 (16) |
|  | TP Correction | -8.89 (1.46) | 0.11 (3.73E-2) | -4.89E-4 (2.81E-4) | 909 (16) |
|  | ComBat | -7.39 (1.46) | 8.71E-2 (3.73E-2) | -3.78E-4 (2.81E-4) | 882 (16) |
| Lateral Ventricles | Raw | 0.38 (0.18) | -8.25E-3 (4.53E-3) | 1.03E-4 (3.40E-5) | 4.29 (1.93) |
|  | TP Correction | 0.37 (0.18) | -8.00E-3 (4.51E-3) | 1.01E-4 (3.40E-5) | 4.32 (1.92) |
|  | ComBat | 0.36 (0.17) | -6.55E-3 (4.38E-3) | 8.50E-5 (3.30E-5) | 3.65 (1.86) |
| Thalamus | Raw | -1.59E-2 (2.41E-2) | -8.45E-4 (6.17E-4) | 5.00E-6 (5.00E-6) | 13.54 (0.26) |
|  | TP Correction | 9.11E-3 (2.35E-2) | -1.162E-3 (6.00E-4) | 6.00E-6 (5.00E-6) | 13.12 (0.26) |
|  | ComBat | -1.26E-2 (2.35E-2) | -7.63E-4 (6.00E-4) | 4.00E-6 (5.00E-6) | 13.39 (0.26) |
| Caudate | Raw | -3.69E-2 (7.34E-3) | 1.12E-4 (8.5E-5) |  | 8.59 (0.13) |
|  | TP Correction | -3.28E-2 (7.33E-3) | 7.60E-5 (8.4E-5) |  | 8.51 (0.13) |
|  | ComBat | -3.04E-2 (7.32E-3) | 5.90E-5 (8.4E-5) |  | 8.43 (0.13) |
| Putamen | Raw | -1.32E-2 (2.01E-2) | -5.64E-4 (5.14E-4) | 4.00E-6 (4.0-6) | 9.79 (0.22) |
|  | TP Correction | -1.33E-2 (2.01E-2) | -5.57E-4 (5.13E-4) | 4.00E-6 (4.0E-6) | 9.79 (0.22) |
|  | ComBat | -1.99E-2 (1.99E-2) | -3.68E-4 (5.09E-4) | 2.00E-6 (4.0E-6) | 9.83 (0.22) |
| Globus Pallidus | Raw | -1.46E-2 (6.15E-3) | 1.53E-4 (1.57E-4) | -8.58E-7 (1.0E-6) | 2.69 (0.07) |
|  | TP Correction | -1.09E-2 (6.18E-3) | 9.2E-5 (1.58E-4) | -5.50E-7 (1.0E-6) | 2.65 (0.07) |
|  | ComBat | -1.11E-2 (6.15E-3) | 9.8E-5 (1.57E-4) | -5.77E-7 (1.0E-6) | 2.63 (0.07) |

TP = Traveling Phantom; Bold white matter age and constant parameters indicates significant difference from raw (p=0.023 and p=0.001, respectively.)
